# Musculoskeletal (Mal)adaptations in Response to a > 30 000‐km Running Challenge

**DOI:** 10.1002/jcsm.70368

**Published:** 2026-08-28

**Authors:** Tomas Venckunas, Thomas Chaillou, Leonardo Cesanelli, Danguole Satkunskiene, Saulius Rutkauskas, Audrius Snieckus, Petras Minderis, Darja Nikitina, Jurgita Skieceviciene, Juozas Kupcinskas, Lorenz Webersberger, Johanna T. Lanner, Mantas Mickevicius, Birutė Gumauskiene, Asta Kuzmickaite, Andrejus Subocius, Sigitas Kamandulis

**Affiliations:** ^1^ Institute of Sport Science and Innovations Lithuanian Sports University Kaunas Lithuania; ^2^ School of Health Sciences Örebro University Örebro Sweden; ^3^ Institute of Metabolic and Cardiovascular Diseases INSERM/Université de Toulouse, Team MetaDiab Toulouse France; ^4^ Department of Radiology Lithuanian University of Health Sciences, Medical Academy Kaunas Lithuania; ^5^ Institute for Digestive Research Lithuanian University of Health Sciences Kaunas Lithuania; ^6^ Department of Gastroenterology Lithuanian University of Health Sciences Kaunas Lithuania; ^7^ Department of Physiology and Pharmacology Karolinska Institutet Stockholm Sweden; ^8^ Department of Physiology, Nutrition and Biomechanics The Swedish School of Sport and Health Sciences Stockholm Sweden; ^9^ Department of Cardiology Lithuanian University of Health Sciences Kaunas Lithuania; ^10^ Sporto Klinika Kaunas Lithuania

**Keywords:** gut microbiota, MHC composition, mitochondrial function, muscle wasting, skeletal muscle, ultra‐endurance

## Abstract

**Background:**

Ultra‐endurance sports are increasingly popular, yet the long‐term physiological consequences of sustained extreme training loads remain poorly understood. In particular, the effects of prolonged ultra‐endurance exercise on skeletal muscle structure, function and molecular remodelling are largely unknown. This case study examined a highly experienced ultra‐endurance athlete who completed a world‐record attempt to run 30 300 km, with extensive phenotyping focusing on skeletal muscle adaptations and recovery.

**Methods:**

A 49‐year‐old male athlete (172 cm, 65 kg) ran ~70 km daily for 15 months. Musculoskeletal, cardiac and visceral ultrasonography, leg muscle strength and power measurements were performed before and after the challenge. Muscle biopsies (*n* = 4) from *vastus lateralis* were obtained immediately after completion and during 17 months of recovery to assess myosin heavy chain (MHC) composition, mitochondrial electron transport chain (ETC) complexes and proteins involved in mitochondrial turnover, autophagy and inflammation. Body composition, haematological and biochemical markers, and gut microbiota composition were monitored longitudinally.

**Results:**

The athlete ran 30 300 km over 444 days, maintaining a daily distance of ~70 km despite substantial musculoskeletal discomfort, including a tibial stress reaction mid‐challenge, which resolved gradually with continued running. Body mass decreased by ~3 kg, primarily reflecting fat loss (~83%), accompanied by reductions in muscle thickness, maximal strength and power. Circulating creatine kinase (3–15‐fold), oxidative stress markers (~50%) and GDF8 (~10%–50%) were sustainedly increased, whereas IGF‐I decreased (~10%–40%), suggesting a reduced anabolic environment during the challenge. Muscle biopsy analyses revealed a progressive recovery of mitochondrial function during the 17 months following the challenge, as evidenced by a progressive increase in ETC protein abundance and the expression of regulators of mitochondrial dynamics and quality control (MFN2, PARKIN, DRP1). In contrast, markers of autophagy, apoptosis and inflammation were decreased during the 17‐months post‐challenge (LC3A/B‐I by ~50%, CASP3 by ~60% and NF‐κB^Ser536^ by ~20%). Muscle fibre composition showed extreme predominance of slow fibres (nearly 100% MHC‐I), which persisted during recovery. Most molecular and functional alterations gradually resolved within 10–17 months. Gut microbiota diversity increased during the challenge, with enrichment of *Bifidobacterium* during running and *Akkermansia* during recovery.

**Conclusions:**

Sustaining daily ultrarunning for more than 1 year induces substantial skeletal muscle remodelling, including reduced muscle size, impaired contractile function and mitochondrial maladaptations, despite largely preserved endocrine and haematological stability. These findings highlight skeletal muscle as a primary physiological system challenged during extreme endurance exercise and demonstrate that recovery from such perturbations may require more than one year.

## 1 Introduction

Regular exercise is associated with substantial and multiple health benefits, and compared with a largely sedentary lifestyle, intense and voluminous training for competitive endurance sports is associated with increased healthy life span [1]^,S1,S2^. At the highest end of the endurance exercise spectrum are multiday ultramarathons, representing a major challenge even for well‐prepared and experienced athletes.

Although ultramarathon runners generally appear healthier than the general population [2], participants of multiday ultra‐endurance challenges can experience severe physiological stress, leading to depletion of energy reserves, loss of functional capacities, and, in some cases, progression toward a pathological state [3, 4]^,S3–S5^. Various physiological and medical aspects of multiday ultra‐endurance events have been investigated, including exercise heart rate [5, 6, 7, 8]^,S6,S7^, energy intake and expenditure [6, 8, 9, 10, 11, 12, 13, 14]^,S6–S15,^ body composition [7, 8, 9, 10, 11, 12, 13, 14, 15, 16],^S8,S9,S12,S14–S18,^muscle strength and power [10, 14, 17, 18], muscle damage [18, 19], muscle fibre type composition^S11,S19^, whole body aerobic capacity [6, 8, 14, 16]^,S11^, metabolic plasticity^S10,S11^, sleep [5, 15]^,S6,S13,S20,S21^, blood glucose, hormones and other circulating markers [6, 8, 10, 14, 20]^,S16^, body core temperature [7], cognition [15]^,S7^, gut microbiota [12] and injuries [21]. Additional parameters have also been examined, such as the cumulative stress response assessed through hair composition^S16^ or dried blood analyses^S12^, as well as muscle enzyme activities and mitochondrial function [14]^,S11^. However, most of these studies lasted less than 3 months, and the long‐term effects of extreme‐volume exercise, particularly on skeletal muscle remodelling, mitochondrial function and gut microbiota, remain poorly understood.

Emerging evidence indicates bidirectional interactions between physical activity and gut microbiota, with endurance athletes showing distinct microbial profiles compared with sedentary individuals [22], ^S22,S23^. Prolonged endurance exercise and extreme training loads have been associated with shifts in gut microbiota composition, potentially influencing energy metabolism, gastrointestinal function and recovery processes^S24^. However, microbiota responses to long‐term extreme endurance challenges remain uncharacterised.

This case study investigated an ultra‐endurance runner attempting to set a world record for the fastest time to run 30 300 km, equivalent to a runnable circumnavigation of the globe. We thus aimed to characterise (mal)adaptations during more than 1 year of self‐supported daily 70 km running and to evaluate the subsequent recovery period. We hypothesised that the ultrarun would induce changes across multiple organ systems, primarily the musculoskeletal system, with active muscles displaying modifications in size and function optimised for extremely prolonged daily contractions but, as a trade‐off, compromising force and power. We anticipated that most of these changes would reverse within several months of recovery after cessation of running. To test the hypothesis, we conducted a comprehensive set of pre‐ and post‐challenge measurements, with particular emphasis on skeletal muscle adaptations at multiple levels, from molecular markers to muscle size and overall function, while also regularly monitoring blood markers and gut microbiota composition throughout the study.

## 2 Methods

### 2.1 The Athlete

A highly experienced ultra‐endurance runner (49 years of age, 172 cm) contacted the research team and expressed interest in having his physiological status monitored by scientists during the upcoming largest athletic challenge of his sports career. He already had an extensive running career before the current challenge (https://www.aidasardzijauskas.com/) and was mentally prepared for the upcoming challenge, with a thorough logistical plan for its execution. The athlete has been repeatedly tested on the treadmill with expired gas analysis over the past decade at the same physiology laboratory. His VO_2_max in recent years was ~58 mL/kg/min (body mass ranged between 66 and 72 kg), and running economy at 12 km/h before the challenge was 210 mL/kg/km, which is average for distance runners [23]. The subject's fastest marathon time of 2:48.00 was achieved at age 35 (i.e., 14 years before the challenge). Resting metabolic rate, measured before the challenge using indirect calorimetry (see Supporting Information for the protocol), was 1502 kcal. Ethical approval from the institutional review board was acquired (#BI‐TRS(B)‐2023‐698). The study was conducted in accordance with the ethical standards of the Declaration of Helsinki. The subject provided oral and written consent for all testing and measurement procedures.

### 2.2 The Challenge

The athlete undertook a self‐supported run to set a new record for the fastest time to cover 30 300 km. Before the challenge, the athlete was running for ~120 km per week, along with doing several hours of resistance and core stability exercises. Due to travel restrictions imposed by the COVID‐19 pandemic during this period, the event took place primarily on streets and in parks of the athlete's hometown (Vilnius, Lithuania; 100–170 m above sea level). Each daily run started at ~5 AM and finished by ~7 PM. The surface was largely asphalt, and the terrain was flat with small overall elevation gain. The athlete was interchangeably wearing several pairs of durable training‐type running shoes of different brands (i.e., not extra lightweight ‘advanced’ highly cushioned, and/or carbon fibre plate‐containing modern shoes). Ambient temperature during the challenge ranged from −22°C to +33°C. During the challenge, to alleviate stiffness and excess fluid accumulation in the legs, the runner had sporadic massages, hot tubs and saunas and frequently morning hot showers. For the same reasons, the athlete also used an intermittent pneumatic gradual compression system (Air Relax PLUS 3.0 Compression Therapy System, Air Relax Inc., United States) bilaterally to the calf or whole leg for some periods in the evenings. After finishing the 30 303‐km ultrarun, physical activity consisted of walking; running was resumed 12 months after and remained restricted to no more than three 1‐h sessions per week until 17 months post‐challenge.

The subject maintained an omnivorous diet before and during the study, and during the challenge, the diet consisted primarily of home‐cooked meals and restaurant dinners rather than sports‐specific products. Along with the normal diet, to meet caloric requirements, the subject consumed carbohydrate‐ and protein‐based sports foods and snacks of various formulations (gels, bars, chocolates, cakes, doughnuts, etc.) at regular intervals during the day. The subject's daily calorie intake during the challenge was analysed independently by two researchers, and the values were then averaged. Based on the runner's food records on the variation in his three typical daily menus (plus supplements) consumed interchangeably and monotonously over the whole period of the challenge, the calculated calorie intake was on average ~5800 kcal/day (chronometer.com): ~45% energy was derived from carbohydrates (~10 g/kg/day, ~50% simple sugars), ~37% from fats (3.7 g/kg/day, ~43% saturated) and ~18% from proteins (4.3 g/kg/day). Caffeine intake was moderate (~2 mg/kg/day) and consumed during the first part of the day. Alcohol consumption was negligible (< 1% of total energy intake). Iron and other minerals (primarily magnesium), vitamins (primarily B_6_, C and D), various amino acids, krill oil, omega‐3 capsules, collagen, creatine, inosine and herbal and plant extracts (e.g., *Tribulus terrestris* , ashwagandha), and some other supplements, all in recommended typical doses for active people and athletes, were taken regularly but not daily. All supplements were discontinued after the challenge.

### 2.3 Measurements

Detailed descriptions of the measurement procedures and methodologies are provided in the Supporting Information. Briefly, body mass was measured; venous blood and stool samples were collected before, approximately once per month during, and several times after the challenge. Anthropometrical, muscle thickness with ultrasound, and muscle force and power measurements were conducted before (PRE), immediately after (POST), and 1 (POST‐1M), 10 (POST‐10M) and 17 months (POST‐17 M) after the challenge. Muscle biopsies were obtained 2 h after the running of the last day of challenge (only 10 km that day), and then at POST‐1M, POST‐10M and POST‐17M. All biopsies were collected in the middle of the day, and POST biopsies were obtained following an overnight fast and without preceding exercise. Viscera and leg ultrasound (Table S1) and echocardiographic examinations were done 3 months before and immediately after the challenge. In addition, during the challenge, an additional examination was performed to investigate the source of leg pain, including ultrasonography of the legs and viscera. Stool samples for microbiome analysis were collected at a single timepoint before (PRE), at eight timepoints during the challenge (1M–15M), at three timepoints during a two‐month recovery period without running (POST 0–2M), and at one time point 10 months after during easy training (POST‐10M) (Table S4). Blood samples were collected at three time points before, 21 timepoints during and 2 months after the challenge. Running statistics were analysed from the Strava account of the runner, who was collecting and uploading data daily using a GPS‐equipped smartwatch (Suunto 9 Baro Titanium Ambassador Edition, Vantaa, Finland). Metabolic scope was estimated from the average energy intake during the challenge divided by the resting metabolic rate measured before the challenge.

## 3 Results

**FIGURE 1:**
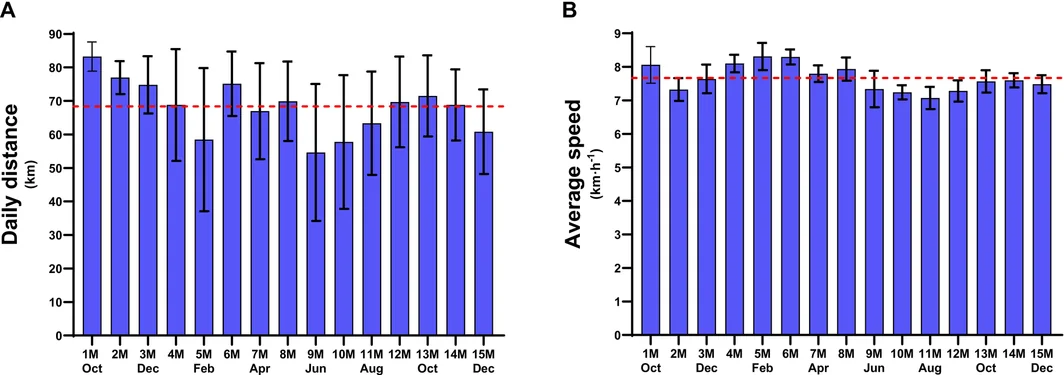
Average daily distances run during the 15 months (M) of the challenge. The error bars represent standard deviations, and the dashed red lines indicate the average daily values over the entire challenge. The names of the months are displayed at 2‐month intervals.

The athlete covered 30 303 km over the span of 444 days (including 4 days off, mainly because of travelling south to continue running there for ~4 weeks during the winter to avoid highly unfavourable homeland weather conditions). Average daily running distance was 68.3 ± 15.7 km · day^−1^ over the entire challenge (Figure 1A), and average running speed of 7.7 ± 0.5 km · h^−1^ was maintained throughout 15 months (Figure 1B). During the first 12 months, the athlete completed 25 032 km (68.6 km · day^−1^). Metabolic scope was sustained at ~3.86 throughout the challenge.

### 3.1 Anthropometrics

Body mass declined during the first few weeks and fluctuated by ~4.5% during the first one‐third of the challenge, then remained relatively constant (Table S3) and was 3 kg lower by the end of the challenge compared with before, followed by an overshoot at 10 and 17 months post‐challenge (Table 1). A similar pattern of changes was observed in skin‐fold thickness‐derived body fat, other anthropometrical measurements and ultrasound‐obtained thigh muscle thickness (Table 1).

**TABLE 1:** Anthropometrics before (PRE), immediately after (POST), 10 months (POST‐10 M), and 17 months (POST‐17 M) after the challenge.

	PRE	POST	POST‐10 M	POST‐17 M
Body mass (kg)	65.0	62.0	70.5	69.6
BMI (kg·m^−2^)	22.0	21.0	23.8	23.5
Body fat (%)	13.0	9.7	17.1	16.1
Circumferences (cm)				
Waist	79	78	83.5	85
Hips	93	92	96	95
Waist/hip ratio	0.85	0.85	0.87	0.89
Chest	98.0	95.0	98.0	99.0
Upper arm	27.5	26.0	27.8	28.5
Lower arm	25.5	23.8	24.8	25.3
Thigh	51.8	47.8	51.5	52.3
Calf	34.5	33.5	35.3	35.3
Muscle thickness (mm)				
Right Vastus lateralis/Vastus intermedius	42.0	39.7	42.9	43.5
Left Vastus lateralis/Vastus intermedius	42.1	39.1	43.6	43.1
Right Rectus femoris/Vastus intermedius	41.0	38.4	45.4	44.6
Left Rectus femoris/Vastus intermedius	41.4	36.2	44.5	44.1

Abbreviations: BMI, body mass index; M, months.

### 3.2 Ultrasonography

There were no major changes in cardiac structure and global function at rest, except for a slight enlargement of left ventricular diastolic diameter (Table S2). No pathological findings were detected in parenchymal organs, except for the slight liver enlargement by the end of the challenge (Table S2).

Several musculoskeletal lesions involving muscles, tendons and ligaments were present in both legs before the challenge, and additional lesions developed or preexisting ones worsened at more than 10 sites during the middle and/or by the end of the challenge (Table S2). However, not all of them were causing pain or discomfort to the athlete. Among the most notable findings were a highly painful tibial stress reaction in the left leg (Figure S1A) and iliotibial band bursitis (Figure S1B), both evident by the midpoint of the challenge. Also, among others, mild tendinosis of the patellar tendon, mild pes anserinus bursitis and mild tendinosis of the semitendinosus tendon with partial tear developed, and tears in both medial menisci became more obvious by the end of the challenge. The thickness of the plantar fascia increased bilaterally from pre‐ to post‐challenge. Overall, the left leg was more affected both at baseline and in response to the challenge (Table S2).

### 3.3 Muscle Force and Power

Vertical jump performance (height and generated power) was 35%–50%, and voluntary and electrically evoked isometric contraction and isokinetic peak torques were ~25% lower at the end of the challenge compared with before (Table 2). Recovery of voluntary and involuntary torques was completed within 10 months, whereas power‐generating ability did not yet return to baseline 17 months after the challenge (Table 2). RSI underwent a particularly large drop from 0.80 to 0.25 after the challenge, remaining well below baseline during the 17 months of recovery. Central activation of the knee extensors was 100% and did not change throughout the study (Table 2).

**TABLE 2:** Neuromuscular function before (PRE) and for up to 17 months (M) after the completion (POST) of the challenge.

	PRE	POST	POST‐1 M	POST‐10 M	POST‐17 M
Jump height (cm)					
SJ	30.3	17.0	18.2	22.1	20.5
CMJ	28.7	15.0	18.5	22.2	23.5
DJ	21.3	10.4	14.5	17.7	22.4
Jump peak power (W/kg)					
SJ	40.6	26.9	30.3	35.7	33.2
CMJ	36.5	23.8	27.3	32.1	31.7
DJ	42.9	21.6	28.8	32.2	33.9
RSI (m · s^−1^)	0.80	0.25	0.40	0.54	0.57
Voluntary torque					
MVIC (right leg), Nm	278	215	226	264	275
CAR (%)	100	100	100	100	100
Right KE‐isoK (60° · s^−1^), Nm	193	159	158	194	200
Left KE‐isoK (60° · s^−1^), Nm	174	131	134	187	193
Right KF‐isoK (60° · s^−1^), Nm	103	78	82	91	90
Left KF‐isoK (60° · s^−1^), Nm	104	48	55	79	88
Right KF/KE	0.53	0.49	0.52	0.47	0.45
Left KF/KE	0.60	0.37	0.41	0.42	0.46
Involuntary torque (right leg)					
P20, Nm	145	120	119	152	153
P100, Nm	199	155	155	209	196
P20/P100	0.73	0.77	0.77	0.73	0.78

Abbreviations: CAR, central activation ratio; CMJ, counter‐movement jump; DJ, drop jump; KE, knee extension; KF, knee flexion; M, months; MVIC, maximal voluntary isometric contraction; P20, P100, torque induced via 20 and 100 Hz supramaximal electrical stimulation; RSI, reactive strength index; SJ, squat jump.

### 3.4 Muscle Proteins and Blood Markers

**FIGURE 2:**
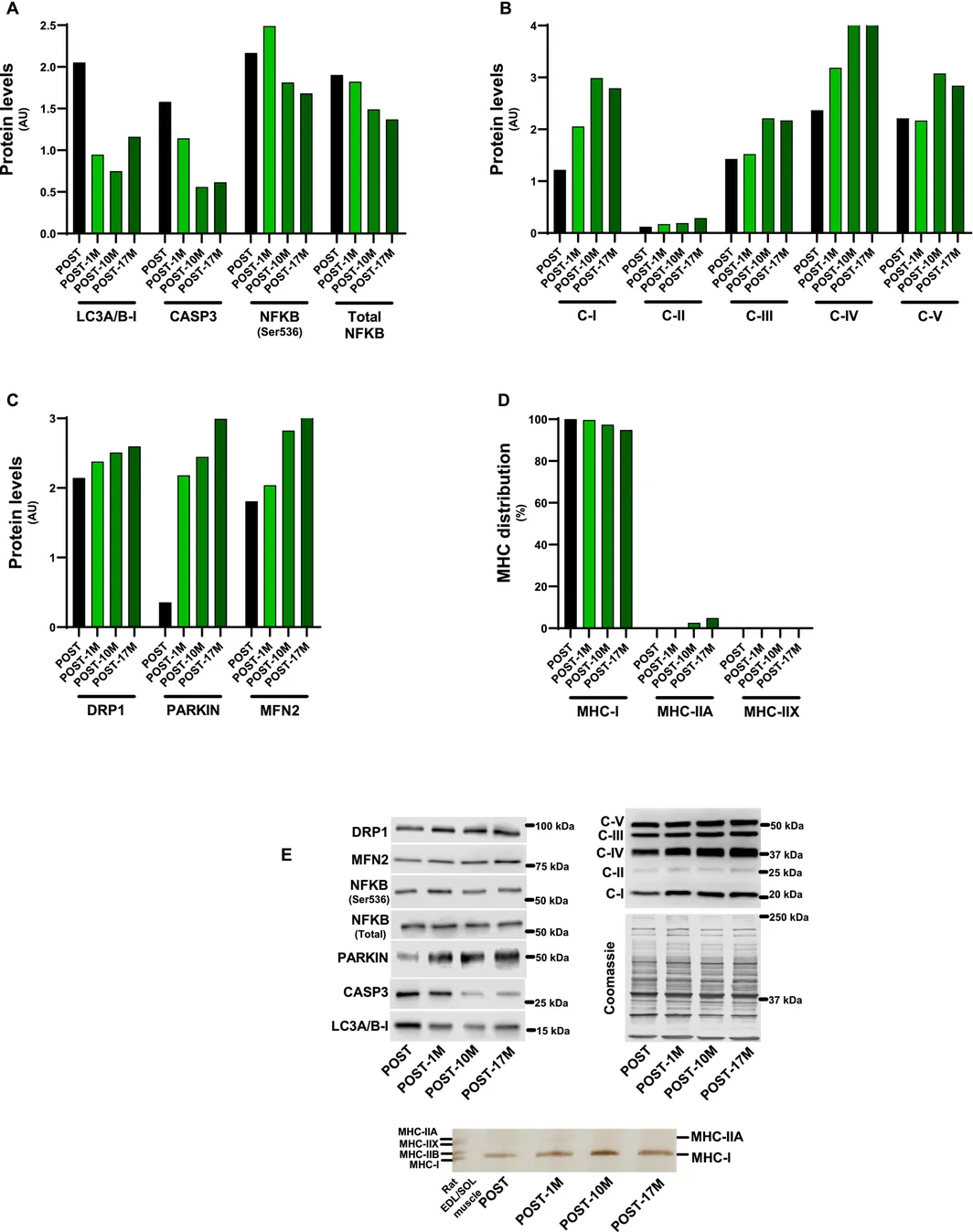
Muscle levels of proteolysis‐related proteins (A), electron transport chain complexes (B), mitochondria‐remodelling proteins (C), and myosin heavy chain (MHC) distribution (D). Representative immunoblots and MHC electrophoresis are shown in E. “Rat EDL/SOL muscle” represents a mixture of extensor digitorum longus and soleus muscles collected from rat. This sample, containing all mature MHC isoforms, confirms the reliability of the procedure. AU: arbitrary units.

Vastus lateralis protein levels of LC3A/B‐I and CASP3 markedly (~50%–60%) decreased from the end of the challenge to 17 months post‐challenge (Figure 2A,E). Similarly, protein levels of total‐ and activated (ser536) NF‐κB p65 decreased (~20%) during the recovery period after the challenge (Figure 2A,E). In contrast, protein levels of ETC complexes (Figure 2B,E) and components of the mitochondrial turnover pathway (DRP1, PARKIN and MFN2) (Figure 2C,E) increased from the end of the challenge to 17 months post‐challenge, with the largest change observed for PARKIN (~8‐fold). MHC composition of m. *vastus lateralis* was almost entirely (≥ 98%) type I after and during the recovery from the challenge (Figure 2D,E).

**FIGURE 3:**
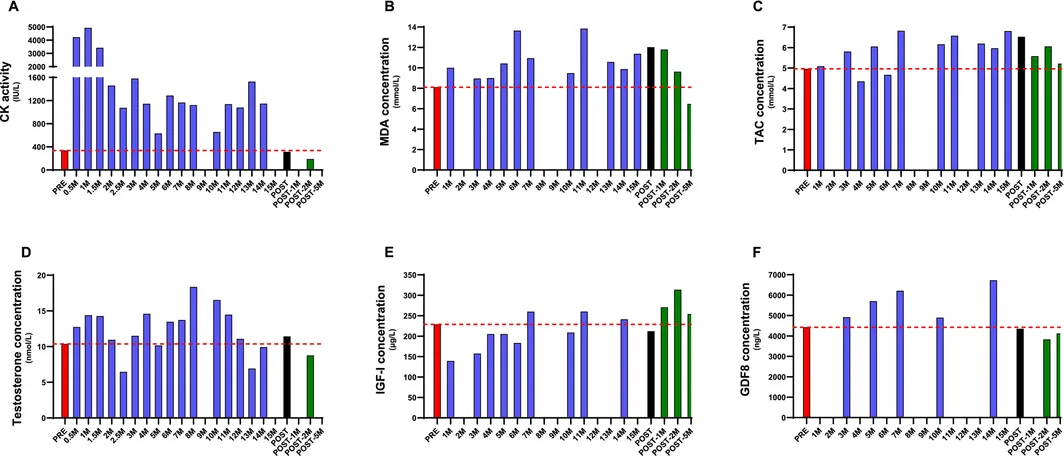
Serum creatine kinase (CK) activity (A), and concentrations of plasma malondialdehyde (MDA, B), plasma antioxidant capacity (TAC, C), serum total testosterone (D), plasma insulin‐like growth factor‐1 (IGF‐1, E), and plasma growth differentiation factor 8 (GDF8, F) before (PRE), during the challenge [1–15 months (M)], immediately after (POST), and during the recovery period (POST‐1M to POST‐5M). The dashed red lines represent the values obtained at PRE.

Serum creatine kinase activity peaked 30 days into the challenge at ~15‐fold above baseline, and then gradually decreased over the following months, but remained ~3‐fold above baseline for the rest of the challenge (Figure 3A). Similarly, serum urea, alanine aminotransferase (ALT) and aspartate aminotransferase (AST) were elevated throughout the challenge above normal levels (Table S3). There was an elevation of both plasma malondialdehyde concentration (a marker of lipid peroxidation) (Figure 3B) and total antioxidant capacity (TAC) of plasma (Figure 3C) during the challenge. Although serum total testosterone concentrations fluctuated throughout the challenge, they generally remained above baseline levels (Figure 3D). Plasma IGF‐1 concentration was overall decreased (Figure 3E), whereas plasma growth differentiation factor 8 (GDF8) concentration increased (Figure 3F) during the challenge. These blood markers returned to baseline values within 2–5 months after the challenge. As shown in Table S3, blood cell counts, haematocrit, haemoglobin concentration, serum creatinine and electrolytes were largely within the normal range and reasonably stable throughout the study. In contrast, ferritin concentration was within the normal range at the start of the challenge (Day 0; Table S3), following a prior iron infusion, but declined over the subsequent weeks. An additional iron infusion was administered approximately 2 months later (day 56 of the challenge), which transiently elevated the ferritin level. However, ferritin remained low throughout the remainder of the challenge despite continued oral iron supplementation, indicating an iron deficiency without overt anaemia (i.e., Hb was preserved). Repeated glucose measurements during routine blood tests revealed values between 3.8 and 5.8 mmol · L^−1^.

### 3.5 Gut Microbiota

**FIGURE 4:**
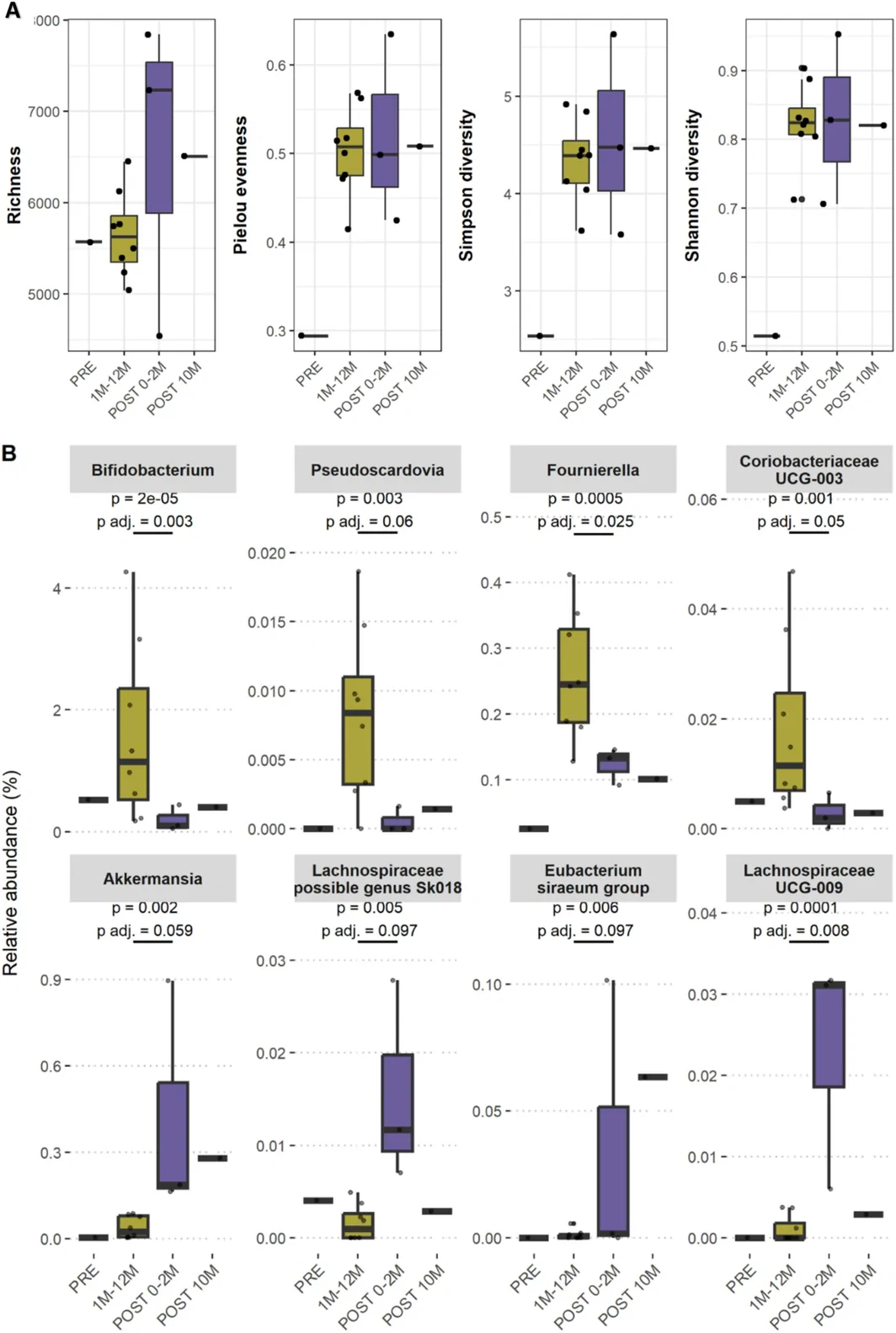
Alpha diversity of stool microbiota from the runner, presented as bacterial richness, Pielou's evenness, Shannon diversity, and Simpson diversity indices (A), and bacterial genera showing the greatest changes in relative abundance across study periods (B). PRE, before the challenge; 1M–12M, months 1–12 of the challenge; POST 0–2M, first 2 months of recovery without running; POST 10M, 10 months after the challenge during gradual return to training.

There was a considerable variation in gut microbiota composition (Figure 4; Figure S2). An increase in bacterial alpha diversity was observed during and after the run when compared with the pre‐run levels (Figure 4). Differential abundance analysis revealed 21 taxa that were significantly different across various taxonomic levels during the run compared with the recovery phase (Table S4). Among these, eight bacteria showed significant differences at the genus level. Genera *Bifidobacterium*, *Pseudoscardovia*, *Fournierella* and *Coriobacteriaceae UCG‐003* were more abundant during the run, whereas *Akkermansia*, *Lachnospiraceae possible genus Sk018*, *Eubacterium siraeum group* and *Lachnospiraceae UCG‐009* were more abundant during the recovery (Figure 4; Table S5). Most of the abundant taxa exhibited consistent differences across multiple taxonomic levels (*Akkermansia*, *Pseudoscardovia*, *Bifidobacterium*, *Fournierella*) (Tables S5 and S6). For instance, *Akkermansia* was more abundant in recovery stool samples not only at the genus level but also at the family, order, class and phylum levels.

### 3.6 Psychological and Medical Issues Encountered

M 1–8: Large psychological stress, acknowledged by the athlete as a major threat to the success of his challenge. It largely arose from social factors, such as health issues affecting a close family member, restrictions imposed during the pandemic lockdown and a conflict at work (firefighting service).

M 2–3: Loss of all toenails due to rainy weather.

M 4–5: Infection of the left big toe developed due to snowy paths, treated with antiseptics.

M 5–6: Ulceration of the pad of the big toe of the left leg. Minor surgical and antiseptic treatment.

M 5–10: Two episodes of severe diarrhoea for several consecutive days.

M 7: A stress reaction of the distal left tibia (Figure S1A) developed slowly until the leg became very painful and swollen, then resolved gradually; no lesions were detectable by the end of the challenge. Iliotibial band bursitis (Figure S1B) developed in both legs during the challenge.

M 9: Episodes of reduced motivation, lack of energy (feeling of debilitation)—a few days of less running.

M 10–14: Amnesia: two episodes of problems in recalling the running route and associated activities for several days in a row.

Respiratory tract infections were avoided, medications were not used and dental repair was not required during the challenge; however, three teeth were lost within 6 months post‐challenge, with periodontitis suspected as the cause.

## 4 Discussion

Average daily distance covered and the average moving speed by our case study participant were comparable with those of the participants of a shorter (4500 km) transcontinental run^S26^. However, our case study participant maintained a daily running distance of nearly 70 km for the longest time ever reported and consequently accumulated 30 300 km over the shortest time so far. Adaptive and some presumably maladaptive changes have been observed in response to the challenge and are discussed below.

### 4.1 Body Mass and Composition

Previous ultra‐endurance running case studies reported modest body mass losses, up to ~1.5 kg, despite daily distances of ~77 km for 28 weeks [11] or ~55 km for 11 weeks [24]. Similarly, our athlete lost ~3 kg (< 5% body mass) during the first 1–2 months, after which body mass remained relatively stable at ~62.5 kg for the remainder of the challenge. This result is consistent with multi‐week ultra‐endurance events such as daily Ironman triathlons^S27,S28^ and ultradistance running [21], in which food intake was sufficient. In contrast, more rapid and pronounced losses occur when energy intake is limited [12, 13, 14, 15]^,S4,S7,S12,S14,S15,S29,S30^. To our knowledge, this is the first study to show that daily ultrarunning for over a year results in a moderate loss of body mass that stabilises within 1–2 months. According to the skin‐fold‐based body fat estimate, our subject lost ~2.5 kg of adipose tissue and only ~0.6 kg of fat‐free mass, which, together with a reduction in segment circumferences (Table 1), suggests a similar reduction of upper and lower limb volume, largely due to fat loss, which was larger than the decline in trunk volume. This pattern resembles sledge‐hauling expeditions, where trunk muscles are better preserved [14] or even hypertrophied [15] compared with arm and leg muscles.

### 4.2 Muscle Damage, Size, Function, Mitochondrial Dysfunction and Fibre Composition

Sustained ultra‐endurance running over more than 1 year imposed profound and chronic stress on skeletal muscle, as reflected by converging biochemical, molecular and functional markers. Serum CK activity peaked 15‐fold above baseline within the first month of the challenge and, although it gradually declined thereafter, it remained ~3‐fold above baseline for the remainder of the event, indicating a persistent increase in myofibre membrane permeability associated with exercise‐induced muscle damage. This could be unfavourable because persistent extracellular Ca^2+^ influx into muscle fibres may activate proteolytic enzymes such as calpains and phospholipases, leading to degradation of cytoskeletal proteins^S31^. Such prolonged elevation is unusual when the training load is stable, albeit high, and implies that muscles are not fully recovering between sessions and that membrane permeability remains chronically increased. Despite persistently elevated markers indicative of ongoing muscle stress and incomplete recovery, the athlete maintained the self‐prescribed running volume and speed throughout the challenge. Similar CK values have been reported during extreme ultra‐endurance races, such as a 1600‐km running [19], but both the magnitude and duration of elevation observed in our case study athlete were substantially greater. Higher CK observed in our runner is likely attributable to both the extreme running challenge and individual susceptibility; however, the present study design does not allow these factors to be disentangled. Regarding the CK time‐course, the marked early increase followed by a decrease to a new plateau of elevated CK may reflect an initial phase of drastic exercise‐induced muscle perturbations during adaptation to the extreme load, followed by a new steady state in which repeated daily bouts of exercise elicit a less pronounced CK response. Concomitant elevations in serum urea and the aminotransferases ALT and AST, which remained 2–3‐fold above baseline throughout the challenge, further support sustained tissue damage, most likely of muscular rather than hepatic origin.

In parallel, systemic oxidative stress was observed. Plasma malondialdehyde, a marker of lipid peroxidation, was elevated during the challenge, alongside an increase in TAC, indicating sustained redox stress and compensatory upregulation of antioxidant defences. Chronic oxidative stress can exacerbate myofibre damage, impair excitation–contraction coupling and interfere with mitochondrial function, potentially linking the extreme mechanical loading of daily ultrarunning to the observed muscle atrophy and functional decline [25]. In contrast, circulating IGF‐1 was reduced at most time points (7 of 10) during the challenge, which, together with elevated GDF8, may have impaired muscle regenerative capacity, thereby amplifying the catabolic effects of mechanical damage and oxidative stress.

Despite this catabolic environment, serum total testosterone remained within the normal range and, on average, above baseline throughout the challenge. This contrasts with reports from polar expeditions and extreme endurance events where salivary or circulating testosterone was markedly suppressed with concomitant substantial body mass loss [15]^,S16,S29^, but aligns with observations that testosterone suppression is not obligatory under prolonged endurance stress [10]. In the present case study, maintenance of testosterone likely reflects the absence of severe or progressive energy deficiency, as body mass loss was moderate and stabilised relatively early during the challenge. This is remarkable, as our case study athlete maintained the largest metabolic scope ever reported in humans for more than 1 year [9]. Oral glucose tolerance test (75 g of glucose) was conducted before the challenge and revealed superior glucose handling and low insulin response (very good insulin sensitivity): This likely helped the runner to tolerate daily consumption of > 600 g of carbohydrate (including > 300 g of simple sugars) to sustain the energy balance.

Voluntary and electrically evoked torque declined markedly, and vertical jump performance exhibited an even larger reduction of up to ~50%, accompanied by similar declines in peak power. Stretch–shortening cycle performance (RSI) was affected even more profoundly and did not fully recover even after 17 months. In contrast, strength markers largely returned to baseline by 10 months of recovery (with fluctuations of only ~10% around baseline). Voluntary activation of the knee extensors remained unchanged throughout, indicating that the observed deficits were predominantly peripheral rather than central in origin, likely reflecting a combination of muscle atrophy, altered tendon compliance and impaired neuromuscular coordination. Measures of the stretch‐shortening cycle showed marked sensitivity to extreme endurance challenges and suggest very prolonged impairment of reactive strength despite MVIC returning to baseline by the 10‐month mark.

The POST muscle biopsy was collected 2 h after completion of the last day run (10 km), a time point chosen partly for practical reasons. We believe that this time point was appropriate to capture both signalling proteins involved in proteolysis and mitochondrial remodelling, as well as structural proteins of the electron transport chain complexes, and to subsequently follow the recovery from that point. The functional impairments after the challenge were accompanied by pronounced skeletal muscle remodelling at the cellular level. Markers of autophagy (LC3A/B‐I), apoptosis (CASP3) and inflammatory signalling (NF‐κB) were higher at the protein content level POST and POST‐1M compared with later in recovery, likely reflecting activation of catabolic pathways and elevated cellular stress. Protein levels of mitochondrial ETC complexes and regulators of mitochondrial dynamics and quality control (MFN2, DRP1, PARKIN) were lowest at POST and then progressively increased, reaching higher levels at POST‐10M and POST‐17M. Although PRE biopsy analysis was missing, these findings may suggest impaired mitochondrial turnover and diminished oxidative capacity at the end of the challenge. These changes presumably contributed to the observed muscle atrophy and weakness.

In our athlete, the extreme predominance of type I fibres (nearly 100% MHC‐I) may reflect a combination of genetic predisposition and long‐term ultra‐endurance training and was likely a critical factor in allowing completion of the challenge of extreme daily running distance. Such a fibre‐type profile, also reported in individual elite endurance runners previously [26], inherently limits rapid power generation, as reflected by persistently low peak power and jump heights.

### 4.3 Musculoskeletal Injuries

The most frequent cause of withdrawal from multiday ultrarunning challenges (e.g., transcontinental races) is musculoskeletal injuries^S25,S26,S32^, and this was considered a major threat to the completion of the challenge in our case study participant. Running‐related injuries are difficult to predict, and a gradual increase in training volume does not appear preventive [27, 28]. Although ultramarathon runners experience a similar overall incidence of exercise‐related injuries compared with shorter‐distance runners, they have a higher proportion of foot stress fractures [2] and a relatively common medial tibial stress syndrome, affecting ~10% of runners [29]. In addition to preexisting lesions, the participant developed or aggravated several injuries during the challenge, most notably semitendinosus bursitis in both legs and a stress reaction in the left tibia midway through the event. Despite medical advice to consider suspending the challenge to avoid progression to a complete fracture, as has been reported in runners who continued training under very high loads [30]^,S33^, he persisted with daily running while managing substantial pain. The pain gradually subsided over several months, and by the end of the challenge, no acute lesions were evident. In his previous challenges, the study participant proved that quite substantial lesions in the legs do not preclude his ultrarunning abilities [31]. Minor lesions were also observed, including thickening of the right Achilles tendon with signs of tendinosis by the end of the challenge. Similar tendon changes after a 4500‐km transcontinental run were not associated with race withdrawal and have been interpreted as potential adaptive responses^S25^. These findings indicate that some musculoskeletal lesions may be inevitable during extremely prolonged endurance challenges, but they do not necessarily preclude completion, particularly in highly motivated ultra‐endurance runners who may benefit from elevated pain tolerance^S34^.

### 4.4 Gut Microbiota

The gut microbiome is increasingly recognised as an important contributor to endurance performance and overall athlete health, influencing energy metabolism, immune regulation and gastrointestinal resilience during prolonged physical stress. Previous work has shown that acute endurance events can rapidly modify gut microbial composition; for example, a marathon [32] and ultramarathon^S35^ running has been associated with an acutely increased abundance of *Veillonella*, a genus that metabolises exercise‐derived lactate and produces short‐chain fatty acids, potentially contributing to improved metabolic efficiency and flexibility. In our case study, we have also seen an increase in bacterial alpha diversity, which has previously been reported with intense endurance training [12]. A considerable variation in gut microbiota composition over the study period could have been related to the runner's condition and diet on the days preceding sampling.

Although microbial richness and diversity were higher during and after the challenge compared with pre‐challenge levels, no significant differences were detected between the running and recovery phases. During the running period, abundance of genera *Bifidobacterium*, *Pseudoscardovia*, *Fournierella* and *Coriobacteriaceae UCG‐003* increased. These taxa are associated with carbohydrate metabolism and short‐chain fatty acid production, suggesting a shift in energy supply during prolonged endurance exercise [22, 32]^,S35,S36^. In contrast, recovery samples were characterised by higher abundances of *Akkermansia*, *Eubacterium siraeum* group and Lachnospiraceae‐related genera. Notably, *Akkermansia* enrichment was consistently detected across multiple taxonomic levels, underscoring the robustness of this shift. Given the established association of *Akkermansia* with gut barrier integrity, metabolic regulation and anti‐inflammatory effects^S37^, its increase during recovery may reflect restoration of intestinal homeostasis following prolonged physiological and metabolic stress^S38^. Overall, our findings suggest that prolonged ultra‐endurance exercise results in stable microbiome adaptations instead of temporary disruptions.

### 4.5 Other Physiological Changes and Impairment of Cognitive Function

A modest ~5% increase in heart mass has been reported after 2 months of daily running over distances similar to those performed by our subject (70 km/day), accompanied by a ~ 5% decrease in body mass [33]. In the transcontinental running case report, no significant changes in cardiac structure and global function have been detected^S32^. These closely resemble the findings of the current study, that is, a slight increase in left ventricular diameter but unchanged mass or other parameters of cardiac structure and function. Plasma volume expansion and decreased haemoglobin concentrations have been documented in response to ultra‐endurance running [7]^,S39^, and fluid retention has been repeatedly documented during multiday ultra‐endurance running [33]^S17,S18^ and in response to simulated Tour de France stages [34]. Although we did not directly measure plasma volume, haemoglobin remained largely within the normal range, body mass remained stable (Table S3) and cardiac chamber volumes did not substantially increase (Table S2), suggesting no substantial haemodilution (plasma volume expansion) during the challenge.

Large training volumes in elite marathon runners can compromise iron status^S40,S41^, and daily running of 55 km for 11 weeks resulted in iron‐deficiency anaemia [24]. Ultradistance races of 100 km can also cause iron loss^S42^. However, during 16 days of daily running of 100 km, serum ferritin increased rather than decreased [35], which should be interpreted as an acute phase effect not related to the iron reserve status [36]. Our athlete began the challenge with high ferritin levels, which declined markedly during the early phase and stayed low throughout the remainder of the challenge despite continued oral iron supplementation, indicating an iron deficiency [37], yet overt anaemia did not develop, as evidenced by stable red blood cell counts and haemoglobin levels (Table S3). Oral iron supplementation continued throughout the challenge, whereas intravenous iron was administered 1 week before and on day 56 of the challenge.

While during the 24‐h or multiday events, circulating glucose has been reported to be maintained [20]^,S39^, extreme expeditions with severe energy deficit, such as Antarctic crossings, can result in deep hypoglycaemia, exhaustion and cognitive impairements^S4^. In our athlete, repeated resting blood glucose readings were 3.8–5.8 mmol/L, and body mass, after the initial drop within the first few weeks, was largely stable throughout the challenge. This, together with other markers such as consistently decreased rather than increased LDL cholesterol levels [38] and haemoglobin level maintained largely within the normal range, suggests that energy intake was sufficient and the state of low energy availability and overt malnutrition were avoided for a large portion of the challenge at least. One of the reasons for the maintained nutritional status could have been a very high protein intake (> 4 g/kg/day). However, the ferritin level, especially during the second half of the challenge, was markedly low, suggesting overall compromised iron status, which could be to some extent inevitable during that sort of arduous running challenge despite continuous iron supplementation. Also, during the second half of the challenge, the athlete experienced memory lapses and decreased motivation to maintain daily mileage due to exhaustion, potentially reflecting cognitive fatigue reported in ultra‐endurance runners^S7,S39^.

### 4.6 Limitations of the Study

The biggest limitation of the study is, inevitably, the single‐participant design, which substantially precludes generalisation of the findings. In addition, several potentially important variables could not be precisely monitored during the challenge, including sleep patterns and changes in running economy. Baseline muscle biopsy was not available, so the impact of the challenge on muscle metabolic and signalling proteins could only be interpreted from recovery measurements. Body composition was assessed by measurement of skinfold thickness (i.e., estimation of subcutaneous fat layer volume) rather than more advanced methods that quantify whole‐body and regional adipose, lean tissue and muscle mass. Likewise, variations in blood markers were possibly to some extent affected by the fluctuations in plasma volume across sampling points. Furthermore, due to the extreme physical and psychological demands of the challenge, the athlete was unwilling to perform exercise tests or donate biopsies during the challenge and refused a post‐challenge treadmill VO_2_max test. Obtaining more stool samples and recording dietary intake before and after the challenge would have strengthened comparisons across the pre‐challenge, running and recovery periods. Finally, detailed dietary assessment during the days preceding sample collection could have helped determine the extent to which changes in dietary patterns contributed to the alterations in the gut microbiome.

## 5 Conclusions

Sustaining approximately 9 h of daily running for over a year appears physiologically feasible for highly motivated, pain‐tolerant individuals with excessive previous running experience and with overall good health and a clear predominance of type I skeletal muscle fibres. Several adaptive changes, including modulation of gut microbiota composition and preservation of endocrine and haematological parameters such as circulating testosterone and haemoglobin, likely supported successful completion. At the same time, multiple potentially maladaptive responses were evident, including reductions in muscle size, contractile capacity and mitochondrial content, underscoring the central role of skeletal muscle as a limiting factor under extreme, prolonged endurance stress.

## Conflicts of Interest

The authors declare no conflicts of interest.

## Supporting information




**FIGURE S1A:** Anteromedial surface of the left distal tibia. Stress reaction of the tibia with periosteal enema (thick arrow), irregular cortex (arrow) and inflammatory response of the surrounding fatty tissue (star). FDL, flexor digitorum longus; TN, tibial nerve; TP, tibialis posterior.

**FIGURE S1B:** Coronal plane of the right lateral knee. Moderate iliotibial band bursitis. LCL, lateral collateral ligament; PT, popliteus tendon.

**FIGURE S2:** Composition of the runner's stool samples for bacterial profiles at the phylum level (A) and genus level (B) PRE, before challenge; 1–12 M, 1 to 12 months of the challenge; POST 0–2 M, during 2 months of recovery without running; POST‐10 M, 10 months after the challenge (easy re‐training).
**TABLE S1:** The checklist of the ultrasound pathological findings.
**TABLE S2:** The ultrasound findings before, during and after the challenge.
**TABLE S3:** Body mass, haematological and blood biochemical indices before, during and after the challenge.
**TABLE S4:** Stool samples included in the microbiome analysis.

**TABLE S5:** Descriptive statistics and differential abundance analysis data of bacterial taxa across the study phases.
**TABLE S6:** Taxonomic classification of bacterial genera identified in the study.

**DATA S1:** Supplementary Information.

## Data Availability

The data that support the findings of this study are available from the corresponding author upon reasonable request.
