# Supplementary material for: Musculoskeletal (Mal)adaptations in Response to a > 30 000‐km Running Challenge
Source: J Cachexia Sarcopenia Muscle. 2026 Aug 28;17(5):e70368. doi: 10.1002/jcsm.70368 (PMC13525328; doi:10.1002/jcsm.70368)
Supplement: Supplementary file 1 [file JCSM-17-e70368-s001.docx]

**Supplemental information**

MEASUREMENTS

***Anthropometrics***

Body mass was measured by using electronic scales (Tanita TBF-300, Japan) in the evenings, 1–2 hours after the completion of the daily running bout and after visiting the toilet. Skinfold thickness was measured at seven sites (triceps, subscapular, chest, midaxillary, suprailiac, abdomen, and thigh) using Harpenden calipers (Baty International Ltd., West Sussex, UK). Body density was calculated using Jackson and Pollock's seven-site equation, and fat mass (%) was estimated from body density using the Siri equation (Jackson & Pollock, 1978). Chest, waist, hips, upper arm, thigh, and calf circumferences were measured using a flexible tape. All measurements were carried out by the same experienced researcher on the participant barefoot and in minimal clothing. Circumferences of the limbs are presented as averages across the left and right sides.

***Neuromuscular testing***

Neuromuscular testing consisted of measurements of electrically evoked torques of the knee extensors as well as maximal voluntary isometric contraction (MVIC) of the knee extensors of the dominant (right) leg, followed by maximal isokinetic concentric contractions of the knee extensors (KE-isoK) and knee flexors (KF-isoK) of the right and then the left leg. During the measurements, the participant was seated upright in a pre-calibrated isokinetic dynamometer chair (System 4; Biodex Medical Systems, Shirley, NY, United States), with shank, trunk, and shoulders stabilized with belts. The shaft of the dynamometer was set at a knee joint angle of 60° (0° = full extension) during MVIC and electrical stimulations, and the knee joint angle ranged between 30° and 100° during KE-isoK and KF-isoK.

The warm-up before the testing consisted of 8 minutes of cycling at 70 rpm on the stationary ergometer (Cardio XT6 BT, Hammer Sport AG, Neu-Ulm, Germany) with the power load set so that the number of watts equaled the participant’s body mass in kilograms. Electrical stimulation was applied using three 12 × 8 cm carbonized rubber surface electrodes (MARP Electronic, Krakow, Poland), lubricated with electrode gel (Medigel, Modi’in, Israel). Two electrodes were placed over the skin in parallel along the heads of the proximal portion of the quadriceps, and the third transversely to cover the distal portion of the quadriceps above the patella. An electrical stimulator (Digitimer DS7A, Digitimer, Hertfordshire, United Kingdom) was connected to the electrodes to deliver 0.5-ms square wave pulses at a constant current of 100 mA and a voltage limit set at 200 V, which fully activates human quadriceps muscles^1^.

Neuromuscular testing began with 1-s electrical stimulations at 20 Hz (P20) and 100 Hz (P100) separated by 3 s. Peak torques were determined, and P20/P100 was calculated.

Then three 5-s attempts of MVIC were performed, separated by 1-min rest. The participant was verbally encouraged to exert maximal effort to reach peak torque as quickly as possible, and a 250-ms test train stimulation at 100 Hz was superimposed when the torque curve was stabilized (visual control by the researcher). Central activation ratio (CAR), a measure of voluntary activation level, was calculated as: (MVIC torque/total peak torque generated with the superimposed stimulation) x 100. The best attempt (with the highest MVIC torque) was selected for analysis.

Finally, after 1 min of recovery, knee concentric extension and flexion at 50%, 70%, and 90% of maximal effort were performed for familiarization before the measurement of concentric force. One minute later, the participant performed three consecutive maximal isokinetic contractions at a low angular velocity (60°/s) for the knee extensors (KE-isoK) and knee flexors (KF-isoK) without interruption. After a 2-minute rest period, the same isokinetic testing procedure, including familiarization, was performed with the left leg.

***Muscle power***

Lower-limb power capability was assessed using the countermovement jump (CMJ), squat jump (SJ), and drop jump (DJ) from a 40 cm box. All jumps were performed on a 102 × 76 cm AMTI AccuPower™-Optimized force platform (Advanced Mechanical Technology, Inc., Watertown, MA, USA) sampling at 500 Hz, using AccuPower Software v4.0. Before testing, the participant received standardized verbal instructions and completed several submaximal familiarization trials to ensure consistent technique. For the CMJ, the participant began from a stable upright stance at the center of the force plate with hands placed on the hips to eliminate the influence of arm swing. The participant was instructed to jump as high as possible using a self-selected countermovement depth. For the DJ, the participant started at the edge of a 40 cm box with hands on the hips and was instructed to step off the box (rather than jump upward), maintain an upright trunk during landing, keep the knees aligned with the toes, minimize ground contact time, and produce maximal vertical propulsion upon take-off, and to land at about the same spot on the plate. For the SJ, the participant began from a static position at the center of the force plate with the knees flexed to approximately 90° and hands placed on the hips. The participant was instructed to jump vertically as high as possible from this position while avoiding any countermovement or trunk-assisted push-off. All trials were monitored in real time. Any jump exhibiting loss of balance, premature countermovement, or obvious technique deviations was discarded and repeated. Three valid trials were recorded for each jump condition (CMJ, SJ, and DJ), with one minute of rest between trials. The highest jump of each mode was used for analysis.

The vertical ground reaction force signal was integrated using the impulse–momentum method to obtain vertical velocity, from which take-off velocity was used to calculate jump height. Vertical displacement was determined by numerical integration of the velocity–time signal. Mechanical power was calculated as the product of vertical ground reaction force and the vertical velocity of the participant’s center of mass. Peak power was calculated across all jump conditions during the concentric phase and normalized to body mass. Reactive strength index (RSI) was calculated as the ratio of DJ height (m) to ground contact time (s).

***Muscle biopsy collection***

Needle biopsies from the non-dominant (left) leg vastus lateralis muscle were collected by a highly experienced surgeon, as previously described^2^. Three biopsy samples (~10 mg each) were collected at each sampling time point. The samples were immediately frozen in liquid nitrogen and stored at − 80 °C until further analysis.

***Western blots***

Muscle samples (~10 mg) were homogenized using a bead shaker (TissueLyser LT, Qiagen) in lysis buffer (20 µL/mg; pH 7.5) containing 50 mM Tris–HCL, 1 mM EDTA, 1 mM EGTA, 10 mM β-glycerophosphate, 50 mM NaF, 0.5 mM sodium orthovanadate, 0,5% triton X-100, and a protease inhibitor tablet (Roche Applied Science, Mannheim, Germany). After centrifugation at 12.000g for 10 min at 4 °C, Total protein concentration was determined using the BCA protein assay (Thermo Fisher Scientific, Massachusetts, USA). Equal amounts of total proteins (5–10 µg) from each sample prepared in Laemmli buffer were heated at 95°C for 5 min (except for the analysis of ETC complexes). Subsequently, the samples were loaded in duplicates onto a 4-15% gradient gel before running the electrophoresis (MiniProtean3 system; Bio-Rad Laboratories, Hercules, USA) for 15 minutes at 50V and 35–70 min at 200 V. The proteins were then transferred on a polyvinylidene fluoride membrane for 1 hour at 100 V. The membranes were incubated for 1 h at room temperature with a blocking solution containing 5% non-fat dry milk powder. Afterwards, the membranes were incubated with primary antibodies from abcam (DRP1, ab184247, 1/1000; PARKIN, ab77924, 1/1000), Cell Signaling Technology (LC3A/B, #4108, 1/1000; CASP3, #9662, 1/1000; NF-κB p65, #8242, 1/1000; NF-κB p65 Ser536, #3033, 1/1000; MFN2, #9482, 1/1000), or from Thermo fisher (ETC complexes, #45-8199, 1/1000) diluted in TBS-T with 5% BSA for 2 h at room temperature or overnight at 4 °C. After three washes of 10 min each with TBS-T, the membranes were probed with anti-rabbit (#7074, 1/3000) or anti-mouse (#7076, 1/3000) secondary horseradish peroxidase-conjugated antibodies (Cell Signaling Technology) diluted in TBS-T with 2.5% non-fat dry milk for 1 h at room temperature. Next, the membranes were washed again with TBS-T (3x 5 min) and then treated with chemiluminescent horseradish peroxidase substrate (#926-95000, LI-COR Biosciences, Lincoln, USA). Digital images were captured using a C-DiGit Blot Scanner and then quantified using Image Studio (LI-COR Biosciences). Finally, the membranes were stained with Coomassie, scanned, and quantified with Image Studio to control for equal loading. For data analysis, duplicate measurements were averaged, all time points were normalized to Coomassie staining, and expressed in arbitrary units.

***Distribution of myosin heavy chain (MHC) isoforms***

MHC isoform distribution was assessed in 5–10 mg of frozen muscle, as previously described^3^. In brief, muscle samples were homogenized in ice-cold lysis buffer (20 µL/mg; pH 7.6) containing 20 mM Hepes, 150 mM NaCl, 5 mM EDTA, 25 mM KF, 1 mM Na_3_VO₄, 5% glycerol, 0.5% Triton X-100, and protease inhibitors. Protein concentration was determined by BCA assay. Lysates were diluted in loading buffer, heated at 95°C for 10 min, and 150 ng of protein were loaded into a gel. The separating gel consisted of 30% glycerol, 8% acrylamide, 0.16% bisacrylamide, 0.2 M Tris-HCl (pH 8.8), 0.1 M glycine, 0.4% SDS, 0.05% Temed and 1% ammonium persulfate. The stacking gel consisted of 30% glycerol, 4% acrylamide, 0.08% bisacrylamide, 70 mM Tris-HCl (pH 6.8), 4 mM EDTA, 0.4% SDS, 0.1% Temed and 1% ammonium persulfate. The gels were run at 4°C during the whole migration period, including 40 min at 10 mA and 23 h 20 min at 140 V. The gels were silver stained, scanned with an office scanner (CanoScan 9000F, Canon, The Netherlands, Amsterdam) and analyzed with UN-SCAN-IT software (version 6.1, Silk Scientific Corporation, Orem, USA). The distribution of MHC isoforms was quantified using positive lane analysis and background correction (single region background value).

***Ultrasound examinations***

*Thickness of knee extensors*

With the participant supine on a massage table, transverse images of the vastus lateralis, rectus femoris, and vastus intermedius muscle thickness were obtained from each leg using B-mode ultrasonography with a 10–15 MHz linear transducer (Echoblaster 128, UAB; Telemed, Vilnius, Lithuania) with minimal probe pressure application. Muscle thickness was defined as the perpendicular distance between the adipose tissue–muscle and muscle–bone interfaces at the image center. Three transverse images were acquired at each site, and the average was calculated using ImageJ software (Wayne Rasband, NIH, Bethesda, MD, USA). For the vastus lateralis–vastus intermedius composite, probe placement was standardized at the midpoint between the greater trochanter and the lateral femoral epicondyle. Rectus femoris–vastus intermedius composite thickness was assessed at 30%, 50%, and 70% of the distance between the anterior superior iliac spine and the superior pole of the patella, and the thickness across these three measurement sites was averaged.

*Comprehensive whole-body ultrasound examinations*

The ultrasound examination of the whole body was performed using a Mindray M7 Diagnostic ultrasound system (Shenzhen Mindray Bio-Medical Electronics Co. Ltd, China) with a linear L14-6Ns (10-12 MHz) and curvilinear C5-2s (2-5 MHz) transducers. Additionally, the ESAOTE MyLab 9 eXP with linear L4-15 (4-15 MHz) and curvilinear C1-8 (1-8 MHz) probes were used, respectively.

All examinations were performed by the same radiologist, highly experienced in ultrasound assessment of the abdominal and musculoskeletal system. The ultrasound images were stored and later analyzed independently by two radiologists blinded to the identity of the subject. The presence of pathology was confirmed if both radiologists agreed with the diagnosis.

The thyroid, abdominal organs, and joints of the runner were scanned to exclude the main pathological changes, which are listed in Table 1. The tendon degenerative changes or tendinosis were evaluated according to the Ingwersen et al. provided grading system^4^. Grade 1 represents a degenerative area of fibrillar disruption involving under 25 % of the height of the tendon, Grade 2 – involving 25–50% and Grade 3 – involving more than 50 % of the height of the tendon. Pathologies other than those provided in Table 1 were also evaluated and described.

Ultrasound examination of the viscera and both legs was performed before (2020-06), during (2021-04), and after the challenge (2021-12).

Two-dimensional echocardiography was performed before (2020-06) and after the challenge (2021-12) using a GE Vivid 7 system (GE Vingmed Ultrasound AS N-3190, Horten, Norway). The same experienced cardiologist performed echocardiographic recordings and measurements. The average of 3 consecutive cardiac cycles for all parameters was calculated. All ultrasound findings are listed in Table S2.

***Hematological and blood biochemical analyses***

Blood samples were collected after an overnight fast by venous puncture from an antecubital vein into vacuum tubes for whole blood analyses, and plasma and serum harvesting. Whole blood and serum were analyzed immediately, while plasma aliquots were stored at −80 °C until the analysis of malondialdehyde (MDA), total antioxidant capacity (TAC), growth differentiation factor 8 (GDF8), and insulin-like growth factor-1 (IGF-1). Due to the limited volume of collected plasma samples, MDA, TAC, IGF-I, and particularly GDF8 were not analyzed at all time points. Blood cell counts were measured from whole blood, and serum was used for creatine kinase (CK), creatinine, urea, ferritin, testosterone, electrolytes, liver enzymes [alanine aminotransferase (ALT), aspartate aminotransferase (AST)], lipid panel, and vitamin D analysis using routine, automated hospital laboratory systems. Spectrophotometric analysis (Spark 10M Tecan, Männedorf, Switzerland) was conducted for plasma TAC (Elabscience, Ref # BC-K271-M, CV 4.1%), MDA (Ref #E1371Hu, BT Lab, Zhejiang, China), IGF-1 (Ref #MD58011, IBL International, GMBH, Germany) and growth differentiation factor 8 (GDF8) concentration (Ref #DGDF80, R&D Systems Inc., Minneapolis, USA) using colorimetric assay kits following the provided instructions. Samples for GDF8 analysis were activated with Sample Activation Kit 1 (Ref #DY010, R&D Systems Inc., Minneapolis, USA) before measurements. All samples were analyzed in duplicates, and the coefficient of variation of all assays was <10%.

***Resting Metabolic Rate***

Resting O_2_ uptake and CO_2_ production were measured as 5‐s averages using a metabolic cart (Omnical RMR/BMR, Maastricht Instruments, Maastricht, the Netherlands). During the measurements, the room air temperature was 22 °C and the relative humidity 45%. The cart was pre‐warmed and calibrated according to the manufacturer's instructions before the measurement. Measurement was conducted in the fasted conditions in the morning,

with the participant comfortably and calmly lying in a supine position on a padded couch under a transparent canopy for 30 minutes in a quiet, dimly lit laboratory. The first 5 min of the recordings were discarded, and the remaining data were averaged. Data falling within 10% of the coefficient of variation were used for subsequent analysis. Carbohydrate and fat oxidation rates were calculated using classic equations, and total 24‐h energy expenditure was calculated using the energy equivalents of 4.2 kcal for 1 g of carbohydrates and 9.1 kcal for 1 g of fat.

***Gut Microbiota Composition***

*Sample Collection and DNA Extraction for Microbiome Profiling*

A total of 13 stool samples were included in the microbiome analysis, consisting of one sample collected before the challenge (PRE), eight samples during the challenge (1M-10M), three samples during the recovery without running after the challenge (POST 0-2M) and one sample 10 months after the challenge during a period of light retraining (POST 10M) (Table S3). Stool samples were collected into sterile plastic tubes and stored at –80 °C until analysis. Genomic DNA was extracted using the QIAamp Fast DNA Stool Mini Kit (Qiagen) following the manufacturer’s protocol. The purity and concentration of the extracted DNA were assessed using a Qubit fluorometer with the Qubit dsDNA BR assay kit (Thermo Fisher Scientific).

*16S rRNA Sequencing for Microbiome Profiling*

Microbial community composition was assessed through 16S rRNA gene sequencing, targeting the V3-V4 region. PCR amplification was performed using 319F and 806R primers, which contained dual indexes and heterogeneity spacer sequences to optimize sequencing efficiency^5^. Amplification efficiency was confirmed through 2% agarose gel electrophoresis. To standardize DNA input across samples, the amplified DNA was normalized using the SequalPrep Normalization Plate Kit (Thermo Fisher). The normalized samples were pooled into a single sequencing library and quantified using the Collibri™ Library Quantification Kit. The final library was sequenced on an Illumina MiSeq platform (2 × 300 bp, Illumina).

To minimize potential contamination, two negative control samples were included at DNA extraction and PCR preparation steps. Negative controls were processed alongside experimental samples to account for potential reagent-derived contamination.

*Bioinformatics Processing of Microbiome Data*

Following sequencing, FASTQ files were demultiplexed based on unique index sequences^6^. Barcode, heterogeneity spacer, primer, and index sequences were trimmed before further analysis. Microbiome data processing was conducted using the DADA2 and phyloseq pipelines^7,8^. The Decontam package was applied to filter out sequences detected in negative control samples, ensuring the removal of potential contaminants^9^.

Taxonomic annotations were assigned to amplicon sequence variants (ASVs) using the latest SILVA bacterial database, employing a naive Bayesian classifier with a pseudo-bootstrap threshold of 80%. Normalization of microbial abundance data was performed using either the DESeq2 package or by calculating relative abundances (in %)^10^.

*Microbiome Diversity Analysis and Statistical Testing*

Alpha diversity, reflecting the richness, diversity, and evenness of microbial species within each sample, was assessed using Vegan package^11^. Statistical differences between groups were evaluated using the pairwise Mann-Whitney test. Differential abundance testing was performed using DESeq2. To control multiple testing, the Benjamini-Hochberg correction was applied to adjust for the false discovery rate (FDR).

**Fig. S2** Composition of the runner’s stool samples for bacterial profiles at the phylum level (A) and genus level (B)

PRE, before challenge; 1M–12M, 1 to 12 months of the challenge; POST 0–2M, during 2 months of recovery without running; POST-10M, 10 months after the challenge (easy re-training).

**Table S1.** The checklist of the ultrasound pathological findings.

| Anatomical structures | Pathological changes | Description |
| --- | --- | --- |
| Thyroid | The size, shape, and echogenicity are normal; no nodules are seen | Present or not |
| Liver | Normal size and shape, echo pattern is normal, the vascular structures are normal in appearance, and no visible masses. | Present or not |
| Pancreas | The echogenicity of the visualized parts is normal, the duct is not dilated, and no visible masses. | Present or not |
| Spleen | Normal size, the echo pattern is homogeneous, and no visible masses. | Present or not |
| Kidneys | The size and shape of the kidneys appear normal; no visible masses or stones. | Present or not |
| Bladder | The wall thickness is normal; no visible intramural or intracavitary masses. | Present or not |
| Peritoneal cavity | Effusion | Present or not |
| Joint | Effusion | Present or not |
| Tendon | Enthesophyte | Present or not |
|  | Tenosynovitis | Present or not |
|  | Tendinosis | Mild, moderate, or severe (Grade 1–3) |
|  | Tear | Partial or full |
| Ligaments | Knee ligament injury | Grade 1–3 |
|  | Ankle ligament injury | Grade 1–3 |
| Meniscus | Protrusion or extrusion | Present or not |
|  | Peripheral tears | Present or not |
|  | Meniscal cyst | Present or not |

**Table S2.** The ultrasound findings before, during, and after the challenge.

| **Anatomical structures** | **Before the challenge**  **(2020-06-20)**  **Day –103** | **Mid-challenge**  **(2021-04-16)**  **Day 197** | **Immediately after the challenge**  **(2021-12-20)**  **Day 444** |  |
| --- | --- | --- | --- | --- |
| **Heart** | | | | |
| L ventricular diameter, mm | 41 | **–** | 43 |  |
| Intraventricular wall th., mm | 9 | **–** | 9 |  |
| L ventricular post. wall th., mm | 10 | **–** | 9 |  |
| L ventricular mass, g | 122.4 | **–** | 122.0 |  |
| L atrium diameter, mm | 37 | **–** | 37 |  |
| L ventricular ejection fraction, % | 57 | **–** | 59 |  |
| Aortic annulus diameter, mm | 20 | **–** | 21 |  |
| Aortic sinuses diameter, mm | 33 | **–** | 33 |  |
| R ventricular diameter, mm | 39 | **–** | 38 |  |
| R atrium diameter, mm | 42 | **–** | 41 |  |
| E, cm/s | 55 | **–** | 68 |  |
| A, cm/s | 42 | **–** | 54 |  |
| E/A | 1.31 | **–** | 1.26 |  |
| **Viscera** | | | | |
| Thyroid | Unremarkable | No changes | No changes |  |
| Liver | Unremarkable | No changes | R lobe diameter increased from 14.3 to 16.0 cm |  |
| Pancreas | Unremarkable | No changes | No changes |  |
| Spleen | Unremarkable | No changes | No changes |  |
| Kidneys | Unremarkable | No changes | No changes |  |
| Bladder | Unremarkable | No changes | No changes |  |
| Fluid | Unremarkable | No changes | No changes |  |
| **Legs** | | | | |
| ***Hip joint*** | No effusion | No changes | No changes |  |
| Rectus femoris insertion | R – Grade 1 tendinosis, small enthesophytes;  L – unremarkable | No changes | No changes |  |
| Hamstring tendons | R – Grade 1 tendinosis; L – Grade 2 tendinosis with small calcifications | No changes | No changes |  |
| ***Knee joint*** | No effusion | No changes | No changes |  |
| Quadriceps femoris tendon | R, L – Grade 1 tendinosis, small enthesophytes | No changes | No changes |  |
| Patellar tendon | Unremarkable | Unremarkable | R – Grade 1 tendinosis with mild vascularity;  L – unremarkable |  |
| Collateral ligaments | Unremarkable | Unremarkable | No changes |  |
| Pes anserinus | Unremarkable | Unremarkable | R – unremarkable;  L – mild bursitis |  |
| Medial meniscus | R, L – protrusion, small peripheral tears | No changes | R, L – protrusion, obvious peripheral tears |  |
| Iliotibial band | R – unremarkable;  L – mild iliotibial band bursitis | R – **moderate iliotibial band bursitis** (Fig. S1A);  L – severe iliotibial band bursitis | No changes |  |
| Lateral meniscus | Unremarkable | No changes | No changes |  |
| Posterior knee | Unremarkable | R – mild semitendinosus bursitis;  L – unremarkable | R – unremarkable;  L – Grade 2 semitendinosus tendinosis with partial tears |  |
| ***Ankle and foot joints*** | No effusion | R – unremarkable;  L – small effusion in the subtalar joint | R – small ganglion from the subtalar joint;  L – unremarkable |  |
| Peroneus tendons | Unremarkable | R – unremarkable;  L – Grade 1 tendinosis | No changes |  |
| Anterior talofibular ligament | R – unremarkable;  L – Grade 1 ligamentosis | No changes | No changes |  |
| Tibialis anterior | R – Grade 1 tendinosis, mild tenosinovitis;  L – Grade 1 tendinosis | No changes | No changes |  |
| Extensor hallucis longus | Unremarkable | No changes | No changes |  |
| Extensor digitorum longus | Unremarkable | No changes | No changes |  |
| Tibialis posterior | R, L – Grade 1 tendinosis | R – no changes;  L – Grade 3 tendinosis with partial tears | R – no changes;  L – Grade 3 tendinosis with full-thickness longitudinal tears |  |
| Flexor hallucis longus | R – unremarkable;  L – full-thickness tear with muscle fibrosis | R – unremarkable;  L – muscle edema with local **stress reaction** **of the tibia** (Fig. S1B) | No changes |  |
| Flexor digitorum longus | Unremarkable | No changes | No changes |  |
| Achilles tendons | R, L – Grade 1 peritendinitis | No changes | R – Grade 1 tendinosis;  L – no changes |  |
| Plantar fascia | Unremarkable | No changes | Increases the size up to 1.1 mm in both legs (from 3.3 to 4.4 mm) without changes in echogenicity |  |

L, left; R, right; E, peak velocity of early left ventricular filling; A, peak velocity of late left ventricular filling.

**Table S3**. Body mass, hematological and blood biochemical indices before, during, and after the challenge.

| **Before the challenge** | | | | **Challenge days** | | | | | | | | | | | | | | | | | | | | | |  | |  |
| --- | --- | --- | --- | --- | --- | --- | --- | --- | --- | --- | --- | --- | --- | --- | --- | --- | --- | --- | --- | --- | --- | --- | --- | --- | --- | --- | --- | --- |
|  | **day –75** | **day –50** | **day 0** | | **16** | **30** | **44** | **56** | **70** | **90** | **105** | **150** | **170** | **190** | **210** | **230** | **285** | **310** | **330** | **350** | **365** | **375** | **395** | **415** | **444** | | **POST 2M** | |
| Body mass, kg | 67.0 | 66.0 | 65.0 | | 63.0 | 62.7 | 62.0 | 61.5 | 61.4 | 63.0 | 64.2 | 62.4 | 62.5 | 62.4 | 62.3 | 62.4 | 62.5 | 62.1 | 62.0 | 62.0 | 61.8 | 61.5 | 62.2 | 62.0 | 62.0 | | 62.5 | |
| WBC [3.8–10.8], 10^9^/L | 5.26 | 5.22 | 5.98 | | 5.30 | 5.56 | 6.28 | 5.83 | 6.56 | 6.31 | 6.06 | 6.49 | 6.23 | 5.61 | – | 5.67 | 6.03 | 5.67 | 6.18 | 4.90 | 5.61 | 6.43 | 5.53 | 6.13 | 8.11 | | 5.1 | |
| Neutrophils [40–70], % | 61.4 | 51.4 | 59.4 | | 59.6 | 64.7 | 65.3 | 67.3 | **71.0** | 69.8 | 62.3 | 69.5 | 61.6 | 58.7 | – | 59.8 | 55.5 | 61.4 | 60.1 | 58.4 | 65.7 | 72.6 | 65.8 | 62.3 | 72.8 | | 53.3 | |
| Monocytes [2–8], % | 4.2 | 4.5 | 4.5 | | 3.2 | 3.4 | 4.2 | 4.9 | 3.2 | 4.3 | 5.2 | 3.7 | 3.4 | 5.0 | – | 4.6 | 5.5 | 6.0 | 5.0 | 6.5 | 6.2 | 4.4 | 5.2 | 7.0 | 4.7 | | 9.2 | |
| Eosinophils [<5], % | 3.1 | 4.3 | 1.6 | | 3.8 | 3.9 | **5.1** | 3.3 | 3.0 | 3.4 | 2.4 | 2.0 | 3.8 | 2.9 | – | 2.6 | 3.5 | 3.4 | 3.2 | **5.1** | 3.9 | 2.2 | 3.4 | 2.8 | 2.7 | | 2.0 | |
| Basophiles [0.5–1.0], % | **1.2** | 0.7 | 0.7 | | 0.6 | 0.5 | 0.9 | 0.9 | 0.8 | 0.6 | **1.8** | 0.4 | 0.4 | 1.0 | – | 0.7 | 0.8 | 0.5 | 0.6 | 0.6 | 0.7 | 0.6 | 0.5 | 0.7 | 0.2 | | **0.4** | |
| Lymphocytes [20–40], % | 29 | 37 | 33 | | 31 | 26 | 24 | 23 | 20 | 21 | 27 | 23 | 30 | 31 | – | 32 | 35 | 29 | 31 | 29 | 24 | 20 | 25 | 27 | 20 | | 35 | |
| RBC [4.4–6.1], 10^12^/L | 5.1 | 5.4 | 5.7 | | 4.8 | 4.4 | 4.5 | 4.9 | 4.6 | 5.1 | 5.1 | 5.3 | 5.5 | 4.8 | – | 5.1 | 5.3 | 5.0 | 5.0 | 4.6 | 5.1 | 4.76 | 5.07 | 5.04 | 5.5 | | 5.7 | |
| Thrombocytes (150–400], 10^9^/L | 271 | 288 | 294 | | 367 | 328 | **448** | 398 | **430** | 382 | 341 | 262 | 329 | 302 | – | 335 | 359 | 358 | 381 | 319 | 340 | 336 | 341 | 362 | 330 | | 265 | |
| Haemoglobin (138–172], g/L | 146 | 157 | 166 | | 147 | **133** | **129** | 140 | 140 | 145 | 149 | 150 | 158 | 140 | – | 151 | 155 | 146 | 148 | **136** | 145 | 142 | 153 | 147 | 157 | | 160 | |
| Hematocrit [41–50], % | 44 | 47 | 50 | | 41 | **37** | **40** | 43 | **40** | 46 | 44 | 45 | 48 | 42 | – | 45 | 45 | 43 | 43 | **39** | 44 | 42 | 43 | 45 | 48 | | 47 | |
| Ferritin [12/35–300/500], µg/L | 35 | **23** | 216 | | 156 | 91 | 45 | **29** | 80 | **34** | **28** | 35 | 42 | **29** | **29** | 38 | **29** | **27** | **19** | **28** | **25** | **24** | **12** | **17** | **19** | | 47 | |
| Creatinine [65–120], µmol/L | 89 | 93 | 96 | | 78 | 70 | 67 | 66 | 66 | 69 | **61** | 75 | 67 | **61** | – | – | 70 | 65 | 74 | 65 | **62** | **60** | 78 | 66 | 67 | | 79 | |
| Urea [2.1–8.5], mmol/L | 7.1 | 7.2 | 8.4 | | **12.0** | **12.3** | **11.0** | **10.6** | **12.3** | **9.7** | **10.8** | **8.8** | **10.5** | **9.5** | – | – | **9.9** | **11.0** | **10.9** | **9.9** | **9.6** | **9.6** | **10.8** | **13.8** | 5.0 | | 6.2 | |
| Total chol [<5.0], mmol/L | **5.3** | – | **6.3** | | 4.3 | 4.4 | 4.8 | 5.2 | 5.0 | **6.0** | **5.5** | 4.7 | **5.7** | **5.2** | – | – | **5.4** | **5.3** | **5.3** | 4.3 | 4.7 | 4.4 | **5.5** | 4.7 | **5.8** | | **6.2** | |
| LDL chol [<3.0], mmol/L | 2.9 | – | **3.6** | | 1.8 | 1.9 | 1.8 | 2.2 | 2.1 | 2.3 | 2.7 | 2.1 | 2.7 | 2.5 | – | – | 2.6 | 2.5 | 2.6 | 2.0 | 2.1 | 1.4 | 1.8 | 2.2 | 2.9 | | **3.8** | |
| HDL chol [>1.0], mmol/L | 1.9 | – | 1.9 | | 2.2 | 2.1 | 2.3 | 2.4 | 2.2 | 2.3 | 2.3 | 2.0 | 2.2 | 2.4 | – | – | 2.2 | 1.9 | 2.3 | 1.9 | 2.0 | 2.1 | 2.2 | 2.2 | 1.9 | | 1.2 | |
| Potassium [3.2–5.2], mmol/L | – | 5.1 | 4.2 | | 4.6 | 4.8 | 4.9 | 4.7 | 4.6 | 5.2 | 4.0 | 4.1 | 4.6 | 4.8 | – | – | 4.1 | **5.7** | 4.9 | 3.7 | 4.8 | 4.4 | 4.4 | **5.7** | 4.6 | | 4.8 | |
| Sodium [135–145], mmol/L | – | 142 | 144 | | 143 | 143 | 142 | 142 | 144 | 142 | 143 | 145 | 143 | 144 | – | – | 144 | 141 | 142 | 139 | 141 | 144 | **147** | 145 | 142 | | 142 | |
| Magnesium [0.7–0.9], mmol/L | 0.85 | 0.90 | 0.79 | | 0.78 | 0.76 | 0.80 | 0.84 | 0.80 | 0.80 | 0.79 | 0.84 | 0.87 | 0.90 | – | – | **0.91** | 0.77 | 0.83 | 0.89 | 0.81 | 0.86 | 0.78 | 0.81 | 0.86 | | **0.92** | |
| Iron [15–200], ng/mL | 25.7 | 15.4 | 20.3 | | – | – | – | – | – | – | – | – | 23.3 | 21.8 | – | – | **13.5** | 19.0 | 21.4 | **9.9** | 40.9 | **11.9** | **6.8** | 15 | **10.3** | | 23.6 | |
| AST, U/L [8–33] | **34** | – | **37** | | – | – | – | – | **94** | **99** | **93** | **46** | **62** | **92** | **72** | **73** | **47** | **71** | **77** | **63** | **81** | **89** | **63** | **87** | **37** | | 25 | |
| ALT, U/L [7–56] | 37 | – | 34 | | – | – | – | – | **120** | **120** | **130** | 53 | **82** | **116** | **95** | **80** | 50 | 53 | **82** | **68** | **94** | **100** | **74** | **84** | 53 | | 31 | |
| Vit. D, nmol/L [>50–75] | – | – | 65 | | 80 | 71 | 69 | 86 | 111 | 83 | 61 | 82 | 57 | 69 | 58 | 80 | – | 114 | 123 | 125 | 102 | 96 | 80 | 105 | 83 | | 52 | |
|  |  |  |  | |  |  |  |  |  |  |  |  |  |  |  |  |  |  |  |  |  |  |  |  |  | |  | |

WBC, white blood cells; RBC, red blood cells; LDL & HDL chol, low & high density lipoprotein cholesterol; AST, aspartate aminotransferase; ALT, alanine aminotransferase; POST-2M, 2 months after the challenge

*Notes*. Normal resting values are given in brackets. Values outside the normal range are given in bold.

**Table S4**. Stool samples included in the microbiome analysis.

| **Time point** | | **Phase** |
| --- | --- | --- |
| PRE | 48 days before the challenge | Baseline |
| 1M–12M | Day 29 | On the run |
|  | Day 77 | On the run |
|  | Day 119 | On the run |
|  | Day 161 | On the run |
|  | Day 181 | On the run |
|  | Day 292 | On the run |
|  | Day 347 | On the run |
|  | Day 371 | On the run |
| POST 0–2M | Day 2 post challenge | Recovery without running |
|  | Day 9 post challenge | Recovery without running |
|  | Day 66 post challenge | Recovery without running |
| POST 10M | Day 298 post challenge | Easy re-training |

**Table S5**. Descriptive statistics and differential abundance analysis data of bacterial taxa across the study phases.

**Table S6**. Taxonomic classification of bacterial genera identified in the study.

**Tables S5 & S6** are uploaded as spreadsheets in an Excel file.

**Fig S1A.** Anteromedial surface of the left distal tibia. Stress reaction of the tibia with periosteal edema (thick arrow), irregular cortex (arrow), and inflammatory response of the surrounding fatty tissue (star).

FDL, flexor digitorum longus; TN, tibial nerve; TP, tibialis posterior.

**Fig S1B.** Coronal plane of the right lateral knee. Moderate iliotibial band bursitis.

LCL, lateral collateral ligament; PT, popliteus tendon.

**References**

1.	Treigyte V, Chaillou T, Eimantas N, Venckunas T, Brazaitis M. Passive heating-induced changes in muscle contractile function are not further augmented by prolonged exposure in young males experiencing moderate thermal stress. *Front Physiol*. 2024;15:1356488. doi:10.3389/fphys.2024.1356488

2.	Cheng AJ, Chaillou T, Kamandulis S, et al. Carbohydrates do not accelerate force recovery after glycogen-depleting followed by high-intensity exercise in humans. *Scand J Med Sci Sports*. 2020;30(6):998-1007. doi:10.1111/sms.13655

3.	Ramos C, Cheng AJ, Kamandulis S, et al. Carbohydrate restriction following strenuous glycogen-depleting exercise does not potentiate the acute molecular response associated with mitochondrial biogenesis in human skeletal muscle. *Eur J Appl Physiol*. 2021;121(4):1219-1232. doi:10.1007/s00421-021-04594-8

4.	Ingwersen KG, Hjarbaek J, Eshoej H, Larsen CM, Vobbe J, Juul-Kristensen B. Ultrasound assessment for grading structural tendon changes in supraspinatus tendinopathy: an inter-rater reliability study. *BMJ Open*. 2016;6(5):e011746. doi:10.1136/bmjopen-2016-011746

5.	Fadrosh DW, Ma B, Gajer P, et al. An improved dual-indexing approach for multiplexed 16S rRNA gene sequencing on the Illumina MiSeq platform. *Microbiome*. 2014;2(1):6. doi:10.1186/2049-2618-2-6

6.	Camarinha-Silva A, Jáuregui R, Chaves-Moreno D, et al. Comparing the anterior nare bacterial community of two discrete human populations using Illumina amplicon sequencing. *Environ Microbiol*. 2014;16(9):2939-2952. doi:10.1111/1462-2920.12362

7.	McMurdie PJ, Holmes S. phyloseq: an R package for reproducible interactive analysis and graphics of microbiome census data. *PLoS One*. 2013;8(4):e61217. doi:10.1371/journal.pone.0061217

8.	Callahan BJ, McMurdie PJ, Rosen MJ, Han AW, Johnson AJA, Holmes SP. DADA2: High-resolution sample inference from Illumina amplicon data. *Nat Methods*. 2016;13(7):581-583. doi:10.1038/nmeth.3869

9.	Davis NM, Proctor DM, Holmes SP, Relman DA, Callahan BJ. Simple statistical identification and removal of contaminant sequences in marker-gene and metagenomics data. *Microbiome*. 2018;6(1):226. doi:10.1186/s40168-018-0605-2

10.	Love MI, Huber W, Anders S. Moderated estimation of fold change and dispersion for RNA-seq data with DESeq2. *Genome Biol*. 2014;15(12):550. doi:10.1186/s13059-014-0550-8

11.	*Vegan: Community Ecology Package*. 2012.
