# Supplementary material for: Musculoskeletal (Mal)adaptations in Response to a > 30 000‐km Running Challenge
Source: J Cachexia Sarcopenia Muscle. 2026 Aug 28;17(5):e70368. doi: 10.1002/jcsm.70368 (PMC13525328; doi:10.1002/jcsm.70368)
Supplement: Supplementary file 2 [file JCSM-17-e70368-s005.docx]

**Supplementary References**

S1. Johansson JK, Kujala UM, Sarna S, Karanko H, Puukka PJ, Jula AM. Cardiovascular health in former elite male athletes. Scand J Med Sci Sports. 2016;26(5):535-543. doi:10.1111/sms.12474

S2. Laine MK, Eriksson JG, Kujala UM, et al. Former male elite athletes have better metabolic health in late life than their controls. Scand J Med Sci Sports. 2016;26(3):284-290. doi:10.1111/sms.12442

S3. Scheer V, Tiller NB, Doutreleau S, et al. Potential Long-Term Health Problems Associated with Ultra-Endurance Running: A Narrative Review. Sports Med. 2022;52(4):725-740. doi:10.1007/s40279-021-01561-3

S4. Stroud MA, Coward WA, Sawyer MB. Measurements of energy expenditure using isotope-labelled water (2H2(18)O) during an Arctic expedition. Eur J Appl Physiol Occup Physiol. 1993;67(4):375-379. doi:10.1007/BF00357638

S5. Stroud MA, Ritz P, Coward WA, et al. Energy expenditure using isotope-labelled water (2H218O), exercise performance, skeletal muscle enzyme activities and plasma biochemical parameters in humans during 95 days of endurance exercise with inadequate energy intake. Eur J Appl Physiol Occup Physiol. 1997;76(3):243-252. doi:10.1007/s004210050243

S6. Berger N, Cooley D, Graham M, Harrison C, Best R. Physiological Responses and Nutritional Intake during a 7-Day Treadmill Running World Record. Int J Environ Res Public Health. 2020;17(16):5962. doi:10.3390/ijerph17165962

S7. Maruff P, Snyder P, McStephen M, Collie A, Darby D. Cognitive deterioration associated with an expedition in an extreme desert environment. Br J Sports Med. 2006;40(6):556-560. doi:10.1136/bjsm.2004.016204

S8. Bircher S, Enggist A, Jehle T, Knechtle B. Effects of an extreme endurance race on energy balance and body composition - a case study. J Sports Sci Med. 2006;5(1):154-162.

S9. Costa RJS, Teixeira A, Rama L, et al. Water and sodium intake habits and status of ultra-endurance runners during a multi-stage ultra-marathon conducted in a hot ambient environment: an observational field based study. Nutr J. 2013;12:13. doi:10.1186/1475-2891-12-13

S10. Hattersley J, Wilson AJ, Gifford RM, et al. Pre- to postexpedition changes in the energy usage of women undertaking sustained expeditionary polar travel. J Appl Physiol (1985). 2019;126(3):681-690. doi:10.1152/japplphysiol.00792.2018

S11. Hyldahl RD, Gifford JR, Davidson LE, et al. Physiological assessment of a 16 day, 4385 km ultra-endurance mountain bike race: A case study. Exp Physiol. 2024;109(2):165-174. doi:10.1113/EP091260

S12. Johnsen BH, Brattebø G, Phillips TM, et al. Crossing the Antarctica: Exploring the Effects of Appetite-Regulating Hormones and Indicators of Nutrition Status during a 93-Day Solo-Expedition. Nutrients. 2021;13(6):1777. doi:10.3390/nu13061777

S13. Lahart IM, Lane AM, Hulton A, et al. Challenges in Maintaining Emotion Regulation in a Sleep and Energy Deprived State Induced by the 4800Km Ultra-Endurance Bicycle Race; The Race Across AMerica (RAAM). J Sports Sci Med. 2013;12(3):481-488.

S14. Paulin S, Roberts J, Roberts M, Davis I. A case study evaluation of competitors undertaking an antarctic ultra-endurance event: nutrition, hydration and body composition variables. Extrem Physiol Med. 2015;4:3. doi:10.1186/s13728-015-0022-0

S15. Reid HAB, Dennison NC, Quayle J, Preston T. Starvation Resulting From Inadequate Dietary Planning for a 50-Day Rowing Expedition. Int J Sports Physiol Perform. 2017;12(8):1012-1016. doi:10.1123/ijspp.2016-0349

S15. Zimberg IZ, Crispim CA, Juzwiak CR, et al. Nutritional intake during a simulated adventure race. Int J Sport Nutr Exerc Metab. 2008;18(2):152-168. doi:10.1123/ijsnem.18.2.152

S16. Gifford RM, Taylor N, Kendall J, et al. Preserved circadian variation in cortisol and androgens during a ski traverse of Antarctica in summer. Sci Rep. 2025;15(1):17726. doi:10.1038/s41598-025-01165-1

S17. Knechtle B, Duff B, Schulze I, Kohler G. A Multi-Stage Ultra-Endurance Run over 1,200 KM Leads to a Continuous Accumulation of Total Body Water. J Sports Sci Med. 2008;7(3):357-364.

S18. Knechtle B, Duff B, Schulze I, Kohler G. The effects of running 1,200 km within 17 days on body composition in a female ultrarunner-Deutschlandlauf 2007. Res Sports Med. 2008;16(3):167-188. doi:10.1080/15438620802104120

S19. Schantz P, Henriksson J, Jansson E. Adaptation of human skeletal muscle to endurance training of long duration. Clin Physiol. 1983;3(2):141-151. doi:10.1111/j.1475-097x.1983.tb00685.x

S20. Nédélec M, Chauvineau M, Guilhem G. On the Road to Camarón: The Sleep of an Ultra-Endurance Athlete Cycling 10,000 km in 24 Days. Int J Environ Res Public Health. 2022;19(8):4543. doi:10.3390/ijerph19084543

S21. Netzer NC, Rausch LK, Gatterer H, Burtscher M, Eliasson AH, Pramsohler S. Extreme sports performance for more than a week with severely fractured sleep. Sleep Breath. 2021;25(2):951-955. doi:10.1007/s11325-020-02172-4

S22. Shalmon G, Ibrahim R, Israel-Elgali I, et al. Gut Microbiota Composition Positively Correlates with Sports Performance in Competitive Non-Professional Female and Male Runners. Life (Basel). 2024;14(11):1397. doi:10.3390/life14111397

S23. Uchida M, Fujie S, Yano H, Iemitsu M. Aerobic exercise training-induced alteration of gut microbiota composition affects endurance capacity. J Physiol. 2023;601(12):2329-2344. doi:10.1113/JP283995

S24. Li Z, Li Y, Wang Y, Chen J, Liu Y. The Athlete Gut Microbiome: A Narrative Review of Multi-Omics Insights and Next-Generation Probiotic Strategies. Nutrients. 2025;17(20):3260. doi:10.3390/nu17203260

S25. Schütz UHW, Schmidt-Trucksäss A, Knechtle B, et al. The TransEurope FootRace Project: longitudinal data acquisition in a cluster randomized mobile MRI observational cohort study on 44 endurance runners at a 64-stage 4,486 km transcontinental ultramarathon. BMC Med. 2012;10:78. doi:10.1186/1741-7015-10-78

S26. Schütz UHW, Schmidt-Trucksäss A, Knechtle B, et al. The TransEurope FootRace Project: longitudinal data acquisition in a cluster randomized mobile MRI observational cohort study on 44 endurance runners at a 64-stage 4,486 km transcontinental ultramarathon. BMC Med. 2012;10:78. doi:10.1186/1741-7015-10-78

S27. Knechtle B, Cuk I, Andrade MS, Nikolaidis PT, Weiss K, Forte P, Thuany M. Case Report: Differences in self-selected pacing in 20, 40, and 60 ironman-distance triathlons: a case study. Front Sports Act Living. 2024 Sep 16;6:1155844. doi: 10.3389/fspor.2024.1155844. PMID: 39351144; PMCID: PMC11439664.

S28. Sousa CV, Pereira RW, Rosemann T, Nikolaidis PT, Knechtle B. Self-Selected Pacing During a World Record Attempt in 40 Ironman-Distance Triathlons in 40 Days. Int J Environ Res Public Health. 2020 Apr 1;17(7):2390. doi: 10.3390/ijerph17072390. PMID: 32244582; PMCID: PMC7177248.

S29. Gagnon, D. D., Pullinen, T., Karinen, H., Rintamaki, H., & Kyrolainen, H. (2011). Recovery of hormonal, blood lipid, and hematological profiles from a North Pole expedition. Aviat Space Environ Med, 82(12), 1110-1117. doi:10.3357/asem.3016.2011

S30. Willmott AGB, Lillis J, Pinto JM, Jewiss M, Turner G, Roberts J. Amateur Rowers to Transatlantic Trio Race Winners: A Case Study Describing the Physiological and Psychological Characteristics, Training Adaptations and Race Responses to Ultra-Endurance Rowing. Eur J Sport Sci. 2025 Sep;25(9):e70040. doi: 10.1002/ejsc.70040. PMID: 40827333; PMCID: PMC12361866.

S31. Zhang BT, Yeung SS, Allen DG, Qin L, Yeung EW. Role of the calcium-calpain pathway in cytoskeletal damage after eccentric contractions. J Appl Physiol (1985). 2008;105(1):352-357. doi:10.1152/japplphysiol.90320.2008

S32. Bhella PS, Kelly JP, Peshock R, Levine BD. Delayed enhancement of the intraventricular septum following an extraordinary endurance exercise. BMJ Case Rep. 2010;2010:bcr0620103096. doi:10.1136/bcr.06.2010.3096

S33. Altuwairqi A. Bilateral Stress Fractures of the Tibia in a Long-Distance Runner. Cureus. 2024;16(10):e70599. doi:10.7759/cureus.70599

S34. Freund W, Weber F, Billich C, Birklein F, Breimhorst M, Schuetz UH. Ultra-marathon runners are different: investigations into pain tolerance and personality traits of participants of the TransEurope FootRace 2009. Pain Pract. 2013;13(7):524-532. doi:10.1111/papr.12039

S35. Grosicki GJ, Durk RP, Bagley JR. Rapid gut microbiome changes in a world-class ultramarathon runner. Physiol Rep. 2019;7(24):e14313. doi:10.14814/phy2.14313

S36. Grosicki GJ, Pugh J, Wosinska L, et al. Ultra-Endurance triathlon competition shifts fecal metabolome independent of changes to microbiome composition. J Appl Physiol (1985). 2023;135(3):549-558. doi:10.1152/japplphysiol.00024.2023

S37. Khalili L, Park G, Nagpal R, Salazar G. The Role of Akkermansia muciniphila on Improving Gut and Metabolic Health Modulation: A Meta-Analysis of Preclinical Mouse Model Studies. Microorganisms. 2024;12(8):1627. doi:10.3390/microorganisms12081627

S38. Wade H, Pan K, Duan Q, et al. Akkermansia muciniphila and its membrane protein ameliorates intestinal inflammatory stress and promotes epithelial wound healing via CREBH and miR-143/145. J Biomed Sci.

S39. Álvarez-Herms J, Jornet K. Physiological Data of Kilian Jornet During the Victory of UTMB 2022: An Exceptional Report of Maximal Metabolical Limits. Sports Med. 2024;54(12):3211-3214. doi:10.1007/s40279-024-02091-4

S40. Berger NJA, Best R, Best AW, et al. Limits of Ultra: Towards an Interdisciplinary Understanding of Ultra-Endurance Running Performance. Sports Med. 2024;54(1):73-93. doi:10.1007/s40279-023-01936-8

S41. Martin DE, Vroon DH, May DF, Pilbeam SP. Physiological Changes in Elite Male Distance Runners Training for Olympic Competition. Phys Sportsmed. 1986;14(1):152-206. doi:10.1080/00913847.1986.11708973

S42. Chiu YH, Lai JI, Wang SH, et al. Early changes of the anemia phenomenon in male 100-km ultramarathoners. J Chin Med Assoc. 2015;78(2):108-113. doi:10.1016/j.jcma.2014.09.004
